# A stitch in the left main artery

**DOI:** 10.1007/s12471-025-01950-7

**Published:** 2025-04-03

**Authors:** Yehia Saleh, Saul Rios, Hussein Shaqra

**Affiliations:** 1 Yehia Saleh, University of Iowa, Iowa, USA; 2 Montefiore Medical Center, New York, USA

A 54-year-old man with a past medical history of a bicuspid aortic valve underwent an aortic valve repair ten years ago. He presented with severe aortic stenosis and aortic root dilatation; therefore, he underwent an aortic root repair with reimplantation of the coronaries and an aortic valve replacement. During the procedure, dense adhesions near the left coronary button were noted, and while excising it, a small perforation occurred in the left main artery, which was immediately repaired. Three days after the operation, he developed an episode of ventricular tachycardia, which resolved spontaneously after two minutes. An echocardiogram showed normal left ventricular function and a well-functioning aortic prosthesis. The coronary angiogram revealed focal stenosis in the mid-left main artery (Fig. 1a and b and videos 1, 2 [see Electronic Supplementary Material]), which was new compared to the preoperative coronary angiogram (Fig. 1c and d and videos 3, 4 [see Electronic Supplementary Material]). The patient subsequently underwent coronary artery bypass grafting (CABG) using the left internal mammary artery to the left anterior descending artery. His postoperative course was unremarkable, and he was discharged home a few days later.

**Fig. 1:**
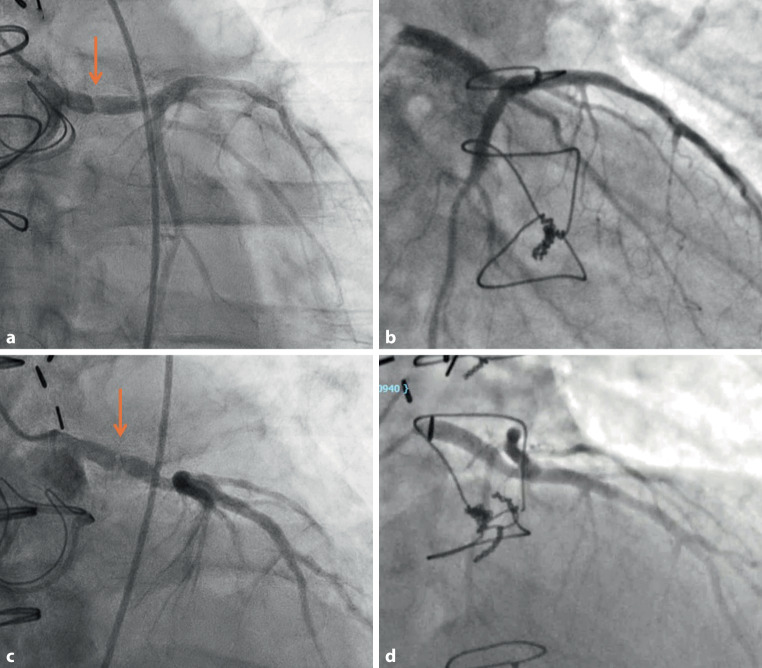
**a**, **b** Postoperative coronary angiogram showing a focal tight stenosis in the mid segment of the left main artery (arrow). **c**, **d** Preoperative coronary angiogram showing a normal left main artery (arrow)

## Supplementary Information

Video 1

Video 2

Video 3

Video 4

